# An improved wrapper-based feature selection method for machinery fault diagnosis

**DOI:** 10.1371/journal.pone.0189143

**Published:** 2017-12-20

**Authors:** Kar Hoou Hui, Ching Sheng Ooi, Meng Hee Lim, Mohd Salman Leong, Salah Mahdi Al-Obaidi

**Affiliations:** Institute of Noise and Vibration, Universiti Teknologi Malaysia, Kuala Lumpur, Malaysia; Tianjin University, CHINA

## Abstract

A major issue of machinery fault diagnosis using vibration signals is that it is over-reliant on personnel knowledge and experience in interpreting the signal. Thus, machine learning has been adapted for machinery fault diagnosis. The quantity and quality of the input features, however, influence the fault classification performance. Feature selection plays a vital role in selecting the most representative feature subset for the machine learning algorithm. In contrast, the trade-off relationship between capability when selecting the best feature subset and computational effort is inevitable in the wrapper-based feature selection (WFS) method. This paper proposes an improved WFS technique before integration with a support vector machine (SVM) model classifier as a complete fault diagnosis system for a rolling element bearing case study. The bearing vibration dataset made available by the Case Western Reserve University Bearing Data Centre was executed using the proposed WFS and its performance has been analysed and discussed. The results reveal that the proposed WFS secures the best feature subset with a lower computational effort by eliminating the redundancy of re-evaluation. The proposed WFS has therefore been found to be capable and efficient to carry out feature selection tasks.

## Introduction

Condition monitoring and fault diagnosis is essential for a wide range of mechanical components to ensure optimal performance. A bearing is a common mechanical component that has an appreciable impact on machine integrity. Vibration signal analysis has been proven to be the most effective method for rotating machinery fault diagnosis. Its effectiveness, however, is highly dependent on the knowledge and experience of the operator [1]. There has been increasing interest in automated machinery fault diagnosis through the adaptive machine learning approach. This provides a more consistent diagnostic outcome; however, the quantity and quality of the input features have a great influence on the fault diagnostic performance. The complexity of the features that have been extracted from a continuous vibration signal leads to the capability of the features remaining unknown, resulting in unconvincing information conversion and representativeness for various conditions, stages or intermediate cycles [2–6]. Meanwhile, an abundance of feature inputs leads to overfitting outcomes. Thus, feature selection is usually performed to identify the most representative feature subsets for the machine learning algorithm to achieve the greatest classification performance by eliminating the overfitting issue [7]. Feature selection is therefore a necessary task to select the most representative feature subsets for the machine learning algorithm.

The feature selection approach can generally be classified into three categories: the filter, wrapper, and embedded methods. Wrapper feature selection alternatives are usually combined with machine learning classifiers to develop a heuristic mechanism that aims to provide an optimal input for targeting optimization functions by considering the options available within a search space boundary. This is performed by the renowned genetic algorithm (GA) [8,9], particle swarm optimization (PSO) [10,11], the ensemble learning algorithm [12], extreme learning machines (ELM) [13], ant colony optimization (ACO) [14,15], the imperialist competitive algorithm (ICA) [16], and the harmony search (HS) algorithm [17,18], among others. This distinctive characteristic gives the wrapper method a much-needed robustness and accuracy, especially with regard to massive, multidimensional data processing, which requires a highly sophisticated classification [19]. Nonetheless, it is obvious that the trade-off relationship between capability in selecting the best feature subset and computational effort is inevitable in the wrapper-based feature selection (WFS) method [20–24]. For instance, the GA involves the iterative identification of a probable solution based on genetic evolution theory. The evaluation resource increases exponentially with regard to the population size and offspring selection strategy. Six extracted features present 63 feature combinations evaluation, while 12 extracted features present 4095 feature combinations for evaluation. Table 1 displays the number of feature combinations for the number of extracted features. It is clear that it would be very computationally demanding for a feature evaluation to be carried out for all feature combinations. Hence, a simplified classification model is beneficial for post-processing system identification, cost-savings and minimizing uncertainty.

**Table 1:** Number of combinations based on the number of features extracted.

Number of features	Number of combinations
3	7
6	63
12	4,095
24	16,777,215
48	281,474,976,710,655

Various feature selection crossover combinations such as the hybrid filter-wrapper method have been implemented, with a twofold aim: To refine the feature selection performance and reduce the disadvantages introduced by individual techniques [25–27]. Nonetheless, the pattern recognition classifier design for real-world cases typically resembles a black box study scheme; it is rather tedious to justify a satisfactory equilibrium among multiple influencing factors without *a priori* knowledge [28]. In addition, overemphasis on either dimension (performance effectiveness or modelling simplification), setting simple algorithm assumptions and overlooking the influence of interrelationships between variables [29] likely jeopardizes the fulfilment of the machine learning objective. As a result, in addition to performing feature selection, a tendency to avoid overdesign in simulation together with sluggishness and premature local optima convergence are equally crucial.

This paper proposes an improved WFS method that aims to select the fittest feature subset with minimum computational resources via selecting potential candidates only through unique feature combinations. This provides the advantage of avoiding the unnecessary consideration of repetitive feature combinations and previously eliminated candidates. In this section, the necessities of the feature selection in automated machinery fault diagnosis and the limitations and drawbacks of the WFS method have been discussed in detail. The methodology for the bearing data collection, from the feature extraction to the proposed selection strategy, is described in the following section. The performance of the proposed WFS method is discussed based on the k-fold cross-validated classifier performance and compared to the recently published Max-Relevance-Max-Distance (MRMD) technique.

## Materials and methods

The following part of this paper describes the methodology of the bearing data collection, the feature extraction and the proposed WFS strategy in greater detail.

### Data collection

**Fig 1:**
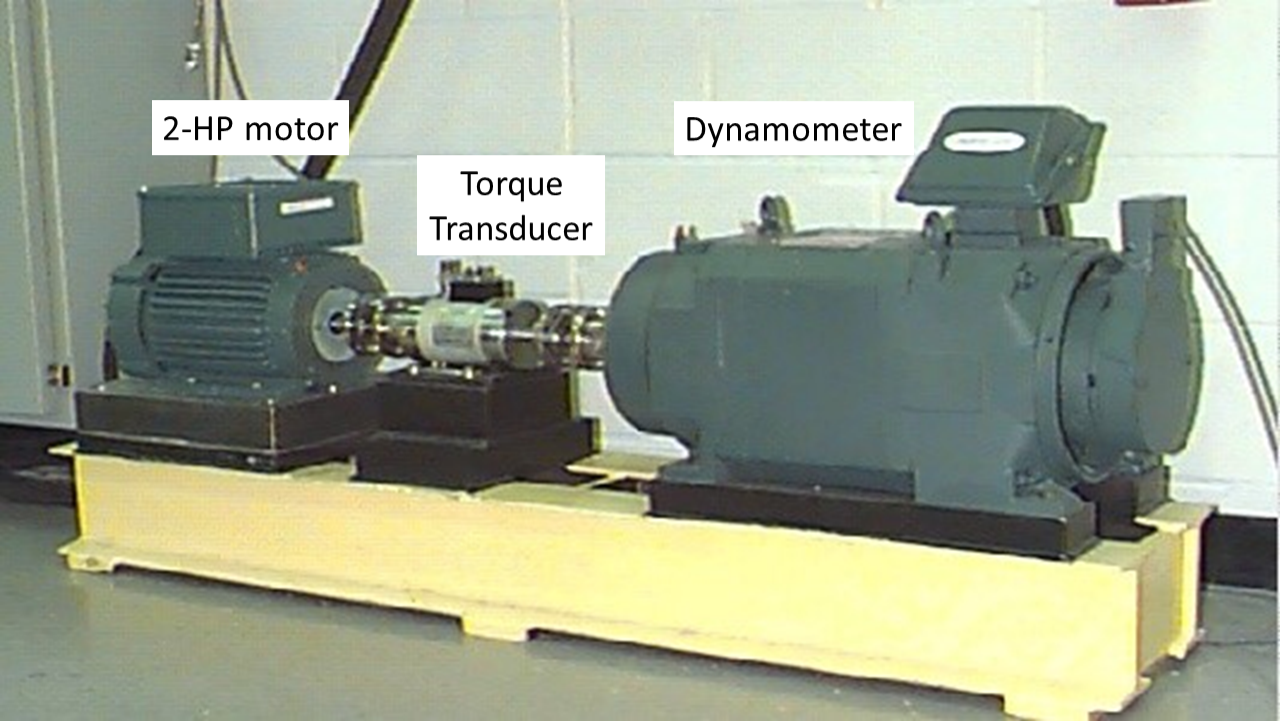
Experimental test rig.

The bearing conditions dataset used in this study was downloaded from the Case Western Reserve University Bearing Data Centre website with the intention of specifically representing ball bearings in healthy and faulty conditions (rolling element, inner raceway and outer raceway faults). The test rig consisted of a 2-horse power (HP) motor, a torque transducer and a dynamometer. The arrangement of the test rig was used to simulate different bearing conditions (Fig 1). The motor operated at approximately 1750 rpm with a 1-HP load. Vibration data were collected at a sampling rate of 12 kHz by accelerometers that were attached to the bearing housing.

A total of 400 sets of time series vibrations were extracted from the raw continuous vibration signal collected from a 7-mil fault diameter with a 1-HP load. Then, the 400 sets of vibration data were divided into two sets of data, one of which was used to establish the relationship between the input and output of the machine learning model (training phase), while the other set was used to validate the trained machine learning model (testing phase). The distribution of the vibration dataset employed in this study is tabulated in Table 2.

**Table 2:** Vibration data distribution.

Bearing condition	Training data	Testing data
Healthy	50	50
Rolling element fault	50	50
Inner raceway fault	50	50
Outer raceway fault	50	50

### Feature extraction

In this section, the time series vibration data from Section 3 is subjected to statistical analyses. The features obtained, namely, the skewness factor, kurtosis factor, crest factor, shape factor, impulse factor and margin factor, were converted from the corresponding equations in Table 3. The statistical features were subsequently used as features (inputs) for SVM model training and testing purposes. Each statistical feature presented has unique characteristics and reveals informative data regarding system status.

**Table 3:** Statistical features.

No.	Statistical Feature	Equation
A	Skewness factor	$\frac{\frac{1}{N}\sum_{n=1}^{N}{\left(x(n)-\bar{x}\right)}^{3}}{{\left(\sqrt{\frac{1}{N}\sum_{n=1}^{N}{\left(x(n)-\bar{x}\right)}^{2}}\right)}^{3}}$
B	Kurtosis factor	$\frac{\frac{1}{N}\sum_{n=1}^{N}{\left(x(n)-\bar{x}\right)}^{4}}{{\left(\sqrt{\frac{1}{N}\sum_{n=1}^{N}{\left(x(n)-\bar{x}\right)}^{2}}\right)}^{4}}$
C	Crest factor	$\frac{max|x(n)|}{\sqrt{\frac{1}{N}\sum_{n=1}^{N}{x(n)}^{2}}}$
D	Shape factor	$\frac{\sqrt{\frac{1}{N}\sum_{n=1}^{N}{x(n)}^{2}}}{\frac{1}{N}\sum_{n=1}^{N}\left|x(n)\right|}$
E	Impulse factor	$\frac{max|x(n)|}{\frac{1}{N}\sum_{n=1}^{N}\left|x(n)\right|}$
F	Margin factor	$\frac{max|x(n)|}{{\left(\frac{1}{N}\sum_{n=1}^{N}\sqrt{\left|x(n)\right|}\right)}^{2}}$

**Fig 2:**
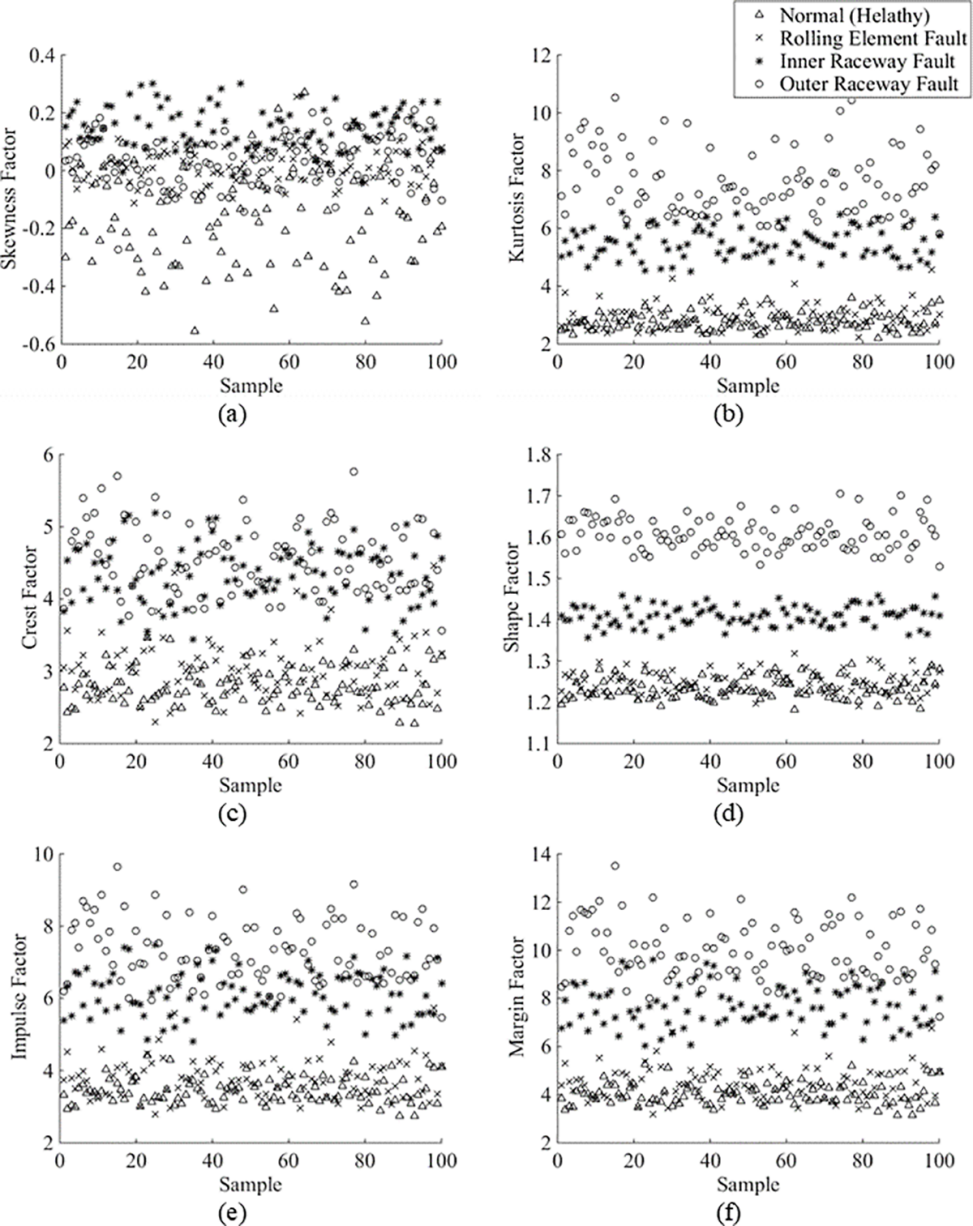
(a) Skewness factor, (b) kurtosis factor, (c) crest factor, (d) shape factor, (e) impulse factor and (f) margin factor of all bearing conditions.

Fig 2 shows the data distribution of the skewness factor, kurtosis factor, crest factor, shape factor, impulse factor and margin factor, respectively, for the vibration signals collected from a 7-mil fault diameter with a 1-HP motor load. The dataset was attached as S1 Data File.

Since there was a total of 100 samples for each bearing condition, 50% of the samples were randomly selected as training data to synthesize the machine learning model, while the remaining 50% of the samples were used to validate the trained machine learning model.

### The proposed wrapper-based feature selection method

**Fig 3:**
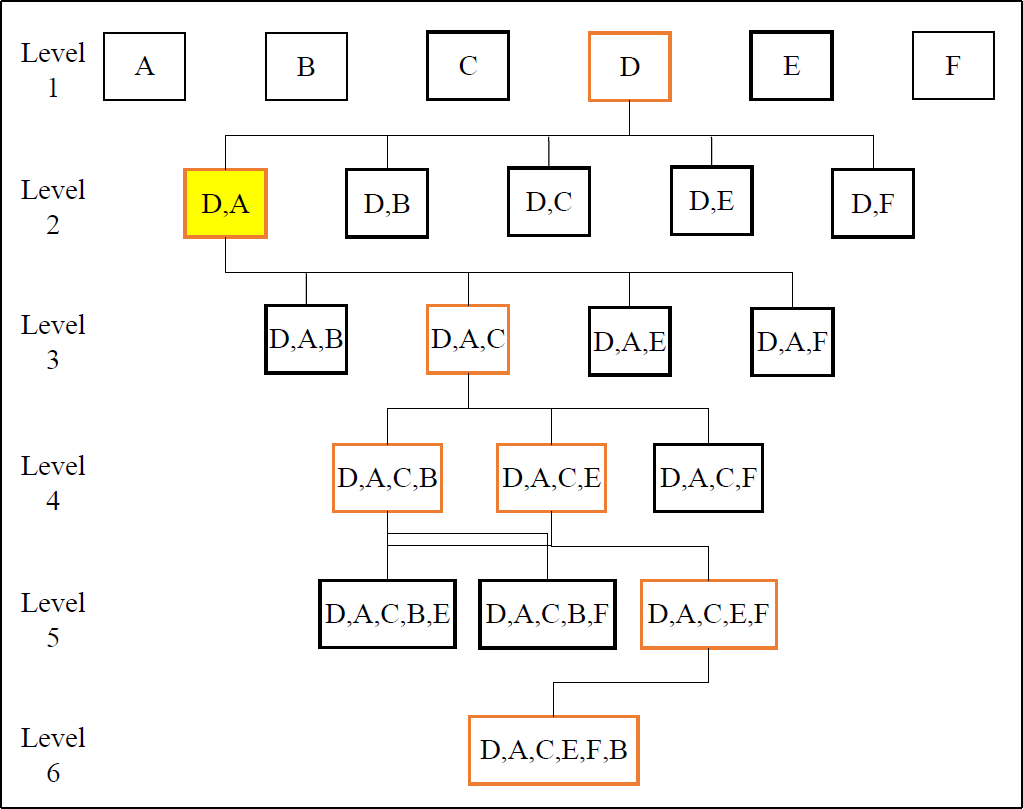
The proposed feature selection algorithm (features A, B, C, D, E and F represent skewness factor, kurtosis factor, crest factor, shape factor, impulse factor and margin factor, respectively).

In this study, an improved WFS method was proposed for performing the feature selection task. The proposed WFS method employed the SVM as a classifier in feature selection. The performance of each feature was based on SVM classifier training accuracy after multi-fold cross-validation appraisal [30] in pursuance of model consistency, by minimizing bias and overfitting. The proposed WFS reduced execution time by avoiding repeated computations of identical and undesirable feature combinations. Thus, for every iteration, the proposed WFS method only evaluated unique combinations of features via two approaches. It is observed by ignoring the repetitive assessment of identical feature combinations that occur during the random generation process of feature combinations and undesirable low quality solutions from past recursive simulation. In addition, the proposed WFS method generated next-level feature combinations based on the performance of the previous level. Fig 3 illustrates the methodology of the proposed WFS algorithm. In first-level selection, the algorithm evaluated each individual feature. Then, the algorithm generated the second-level feature combinations by combining unselected individual features with the features that performed at an above-average level (red-outlined rectangle in Fig 3). This process terminated when the feature combination had fully utilized all the features extracted. Finally, the algorithm selected the feature combinations with the least number of features from the highest training accuracy (yellow-filled rectangle in Fig 3) as the most representative features of the entire dataset. In addition to selecting the most representative features of the dataset, the feature selection also reduced the feature dimensionality for machine learning algorithms. As a result, the skewness factor and shape factor (i.e., features A and D) were selected in this example.

## Results and discussion

Table 4 shows the training accuracy of the key combinations of features at each level. The yellow-shaded feature combinations are those with the best training accuracy at each level, and the blue-shaded training accuracy cell designates the best training accuracy in the table. As a result, features A and D (skewness and shape factor) were selected to represent the entire bearing conditions dataset. The training accuracy in Table 4 indicates that entering all the extracted features into the machine learning algorithm does not guarantee the highest classification accuracy, as the training accuracy for the selected features (i.e., features A and D) was 81%, and the training accuracy for all the features extracted was 74%. In contrast, the testing accuracy of the bearing faults dataset was 83% for the selected features and 76% for all the features extracted. A representative feature combination for the entire dataset was therefore selected using the proposed WFS algorithm.

**Table 4:** Training accuracy for the key combination of features (features A, B, C, D, E and F represent skewness factor, kurtosis factor, crest factor, shape factor, impulse factor and margin factor, respectively).

Level 1		Level 2		Level 3		Level 4		Level 5		Level 6	
Feature	Accuracy	Feature	Accuracy	Feature	Accuracy	Feature	Accuracy	Feature	Accuracy	Feature	Accuracy
A	28.5%	D,A	81.0%	D,A,B	73.0%	D,A,C,B	73.5%	D,A,C,B,E	73.5%	D,A,C,B,E,F	74.0%
B	40.5%	D,B	50.0%	D,A,C	73.5%	D,A,C,E	73.5%	D,A,C,B,F	73.5%		
C	2.5%	D,C	50.0%	D,A,E	72.5%	D,A,C,F	73.5%	D,A,C,E,F	73.5%		
D	50.0%	D,E	50.0%	D,A,F	73.5%	D,A,F,B	72.0%				
E	23.0%	D,F	50.0%			D,A,F,E	73.0%				
F	34.0%										

**Fig 4:**
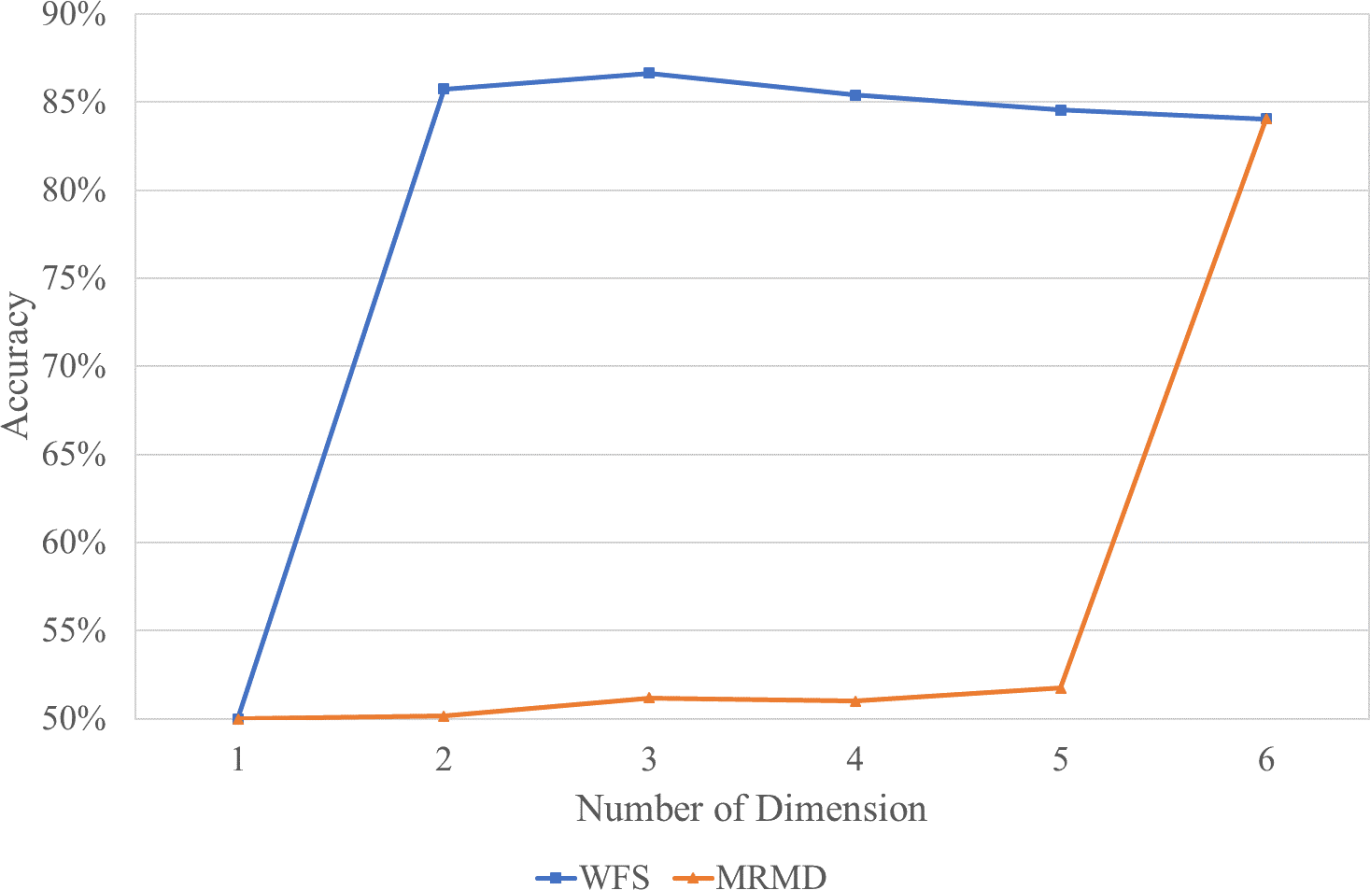
Comparison of the testing accuracy (average of 10-fold cross-validation).

Further investigation has been conducted using a recently published feature selection technique in order to validate the proposed WFS method. The MRMD technique was selected after it demonstrated a good balance between classifier accuracy and stability when subjected to an image processing dataset [31,32]. Its superiority was compared to alternatives such as minimal-redundancy-maximal-relevance (mRMR) [33] and Information Gain. Tables 5 and 6 tabulate the cyclical assessment of the proposed WFS and MRMD. The testing accuracy was obtained through 10-fold cross-validation to represent a more reliable testing result. Fig 4 displays the comparison of the testing accuracy for feature subsets selected by the proposed WFS and MRMD in different dimensions. The proposed WFS became saturated after selecting the second features. Compared to the MRMD, the training accuracy of the WFS is higher until the sixth feature is selected. It is important to acknowledge that the WFS method obtained the optimal feature subset more quickly than the MRMD; however, the latter provides a better consistency in term of classifier outcome when selecting the feature and is more significant when enormous feature subsets are available. This is probably because, initially, the WFS targeted a machinery faults application that supplies limited features while the MRMD aims for an image processing practice.

**Table 5:** Cyclical assessment for the proposed WFS by 10-fold cross-validation.

Cycle	Number of Feature Dimension					
	1	2	3	4	5	6
1	0.500	0.850	0.880	0.815	0.845	0.850
2	0.500	0.870	0.880	0.835	0.880	0.860
3	0.500	0.840	0.870	0.875	0.875	0.865
4	0.500	0.825	0.845	0.860	0.805	0.805
5	0.500	0.885	0.900	0.865	0.745	0.850
6	0.500	0.845	0.845	0.845	0.860	0.845
7	0.500	0.860	0.840	0.885	0.870	0.870
8	0.500	0.860	0.865	0.875	0.860	0.825
9	0.500	0.880	0.860	0.820	0.850	0.835
10	0.500	0.860	0.880	0.865	0.865	0.800
Mean	0.500	0.858	0.867	0.854	0.846	0.841
± SD	± 0	± 0.018	± 0.019	± 0.024	± 0.041	± 0.024

**Table 6:** Cyclical assessment for the MRMD by 10-fold cross-validation.

Cycle	Number of Feature Dimension					
	1	2	3	4	5	6
1	0.500	0.500	0.505	0.500	0.500	0.850
2	0.500	0.500	0.515	0.550	0.500	0.860
3	0.500	0.500	0.500	0.520	0.500	0.865
4	0.500	0.500	0.500	0.500	0.500	0.805
5	0.500	0.500	0.500	0.500	0.520	0.850
6	0.500	0.500	0.500	0.500	0.525	0.845
7	0.500	0.505	0.535	0.500	0.570	0.870
8	0.500	0.510	0.520	0.500	0.500	0.825
9	0.500	0.500	0.545	0.500	0.550	0.835
10	0.500	0.500	0.500	0.530	0.510	0.800
Mean	0.500	0.502	0.512	0.510	0.518	0.841
± SD	± 0	± 0.003	± 0.017	± 0.018	± 0.025	± 0.024

## Conclusion

The aim of this study was to improve the capability of the WFS method for selecting the best feature subset with a reduced computational effort. The analysis of the results revealed that the proposed WFS is capable of selecting the most representative feature subset for the bearing dataset. In addition, this study also confirmed that entering all the extracted features into the machine learning algorithm does not guarantee the best classification performance. Thus, feature selection plays a vital role in ensuring the optimum performance of a classifier. The proposed WFS method also reduces the number of feature combinations needing to be evaluated by avoiding the re-evaluation of identical feature combinations. This reduced the computational effort required by two thirds. In sum, the main advantage of the novel, state-of-the-art WFS method introduced here is its ability to select the best feature subset using less computational effort. This is essential when analysing a large number of inputs. This proposed WFS method should be embedded into machine learning algorithms in order to improve their performance. A further improvement of the proposed WFS method can focus on the selection of image related visual features.

## Supporting information

S1 Data File Dataset for features selection. (MAT)
